# Tumor-to-tumor metastases in Cowden’s disease: an autopsy case report and review of the literature

**DOI:** 10.1186/s13000-015-0408-8

**Published:** 2015-09-17

**Authors:** Karen Matsumoto, Kanae Nosaka, Tatsushi Shiomi, Yuki Matsuoka, Yoshihisa Umekita

**Affiliations:** Division of Organ Pathology, Department of Pathology, Faculty of Medicine, Tottori University, 86 Nishicho, Yonago, Tottori 683-8503 Japan

## Abstract

Tumor-to-tumor metastasis is a rare phenomenon, but it has been suggested to be more frequent in patients with hereditary cancer syndrome. We report an autopsy case of tumor-to-tumor metastasis in a 75-year-old male. At 6 months before his death, the patient complained of hoarseness and dysphagia, and clinical whole-body examinations revealed advanced lung adenocarcinoma (T4N2M1b, Stage IV), multiple skin verrucas, gastrointestinal polyposis, goiters, and cerebellar dysplastic gangliocytoma (Lhermitte-Duclos disease), while *PTEN* gene mutation was detected in his serum. An mTOR inhibitor had been used to treat his lung adenocarcinoma, but he developed aspiration pneumonia and died of respiratory failure. Autopsy revealed that the lung adenocarcinoma had metastasized to cavernous hemangiomas of the right atrial appendage and liver, to cerebellar dysplastic gangliocytoma and to multiple organs such as the liver, kidney, adrenal glands and spine. This is the first reported case of Cowden’s disease with multiple tumor-to-tumor metastases.

## Background

Tumor-to-tumor metastasis (TTM) is a term describing the presence of two histologically distinct tumors at one location, each with different morphologic and immunophenotypic features [1]. The most common metastatic donor neoplasms are lung cancer followed by breast cancer, while meningioma is the most common recipient of metastasis among benign tumors, and renal cell carcinoma is the most common recipient among malignant tumors [2]. It has been suggested that patients with hereditary cancer syndrome may be at risk for the developing TTM [3]. Cowden’s disease is a hereditary cancer syndrome that consists of multiple hamartomas and is associated with increased risks of malignant neoplasms in multiple organs. However, to our knowledge, there has been no report describing TTM in Cowden’s disease. We report herein an extremely rare case of Cowden’s disease with multiple TTM, and discuss the association between TTM and hereditary cancer syndrome.

## Case presentation

### Clinical presentation

**Fig. 1:**
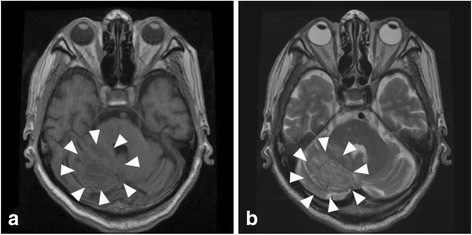
MRI findings in cerebellar dysplastic gangliocytoma. T1 (**a**) and T2 (**b**) weighted magnetic resonance images of the head. A striped pattern is seen in the right hemisphere of the cerebellum (arrowheads). The cerebral aqueduct is compressed

A 75-year-old Japanese male complained of hoarseness and dysphagia. By various whole-body examinations, he was diagnosed with lung adenocarcinoma (T4N2M1b, Stage IV). Also found were a right cerebellar tumor (Fig. 1a and b), dermal multiple verrucas, papillomatosis in the mouth, goiters, and gastrointestinal polyposis. Together these results suggested Cowden’s disease. *PTEN* gene mutation, a point mutation (TGT to CGT) at exon 5 in codon 136, was detected in his serum. Finally, a definitive diagnosis of Cowden’s disease was made. Everolimus, an mTOR inhibitor, was used for 10 days to treat the lung adenocarcinoma but was stopped after aspiration pneumonia began to develop. After several days, he died of respiratory failure.

### Pathology

#### Gross findings

**Fig. 2:**
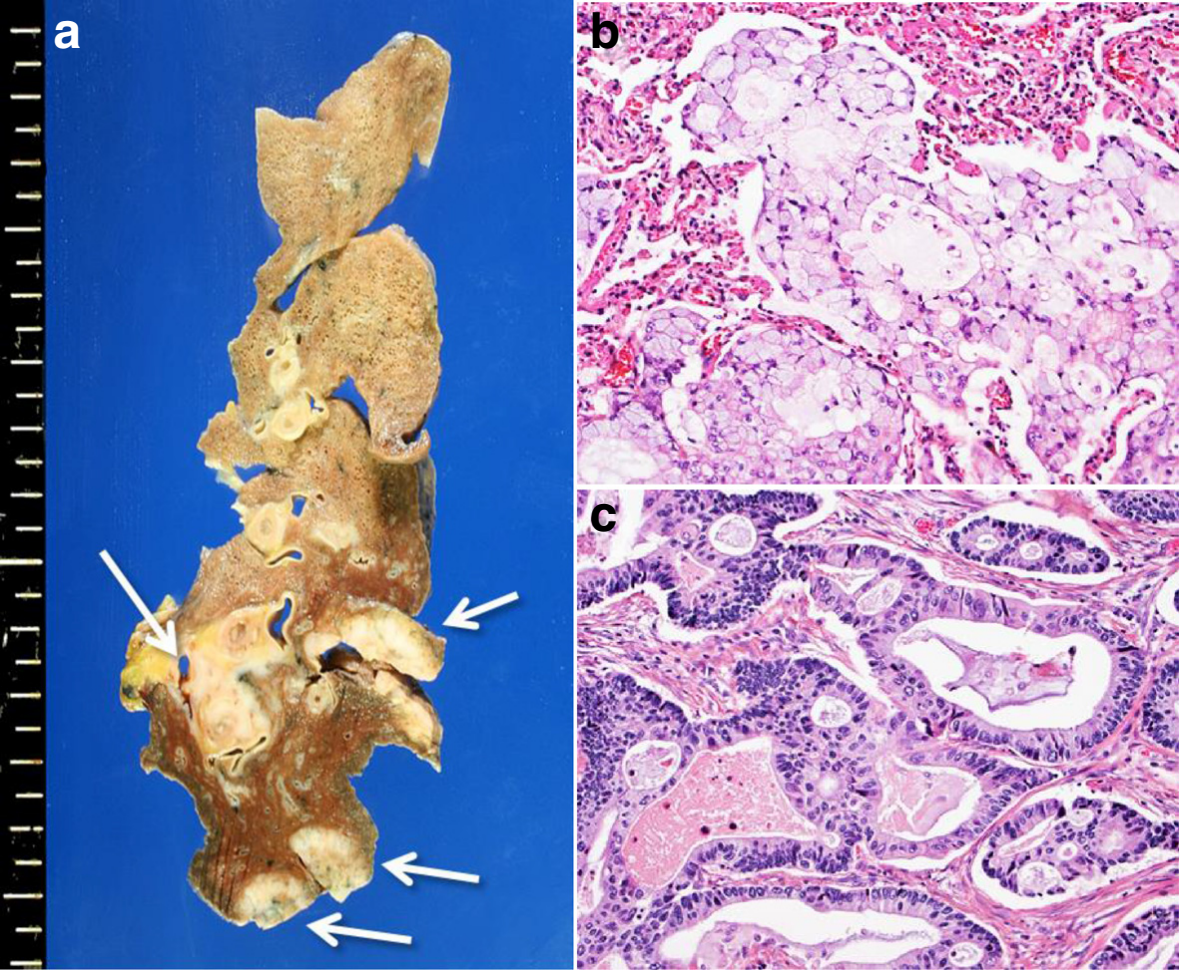
Macroscopic and histologic findings of lung adenocarcinoma. **a** Right lungs showing multiple solid nodules (arrowheads). **b** Solid pattern with signet-ring cells (H.E. x200). **c** Acinar pattern (H.E. x200)

**Fig. 3:**
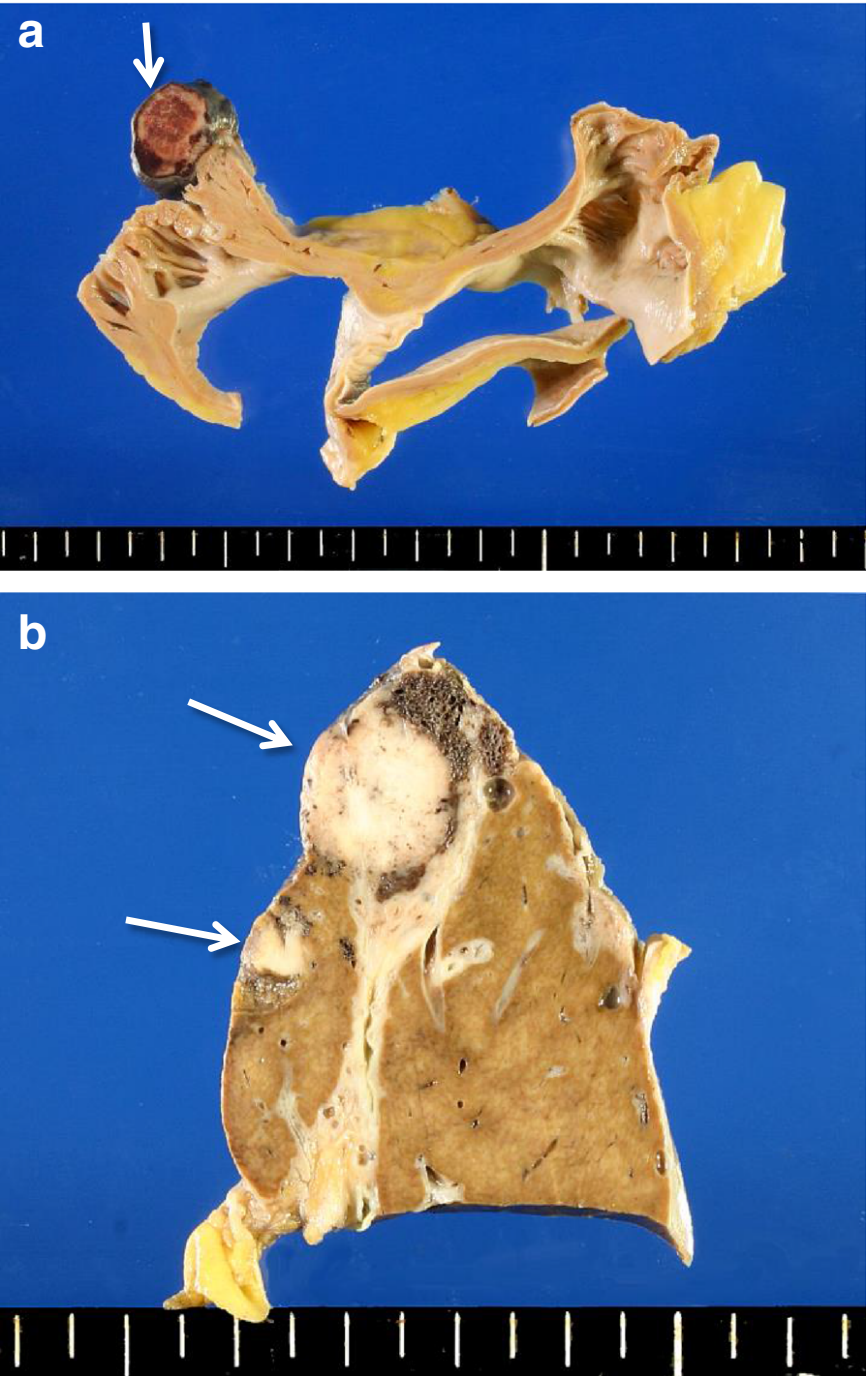
Macroscopic views of tumor-to-tumor metastasis from lung adenocarcinoma to hemangiomas of the right atrial appendage (**a**) and liver (**b**)

Macroscopic examination showed white solid nodules of 10–20 mm in diameter located in S8, S9, and S10 of the left lung and in S7 and S10 of the right lung (Fig. 2a). Well-demarcated reddish nodules in the right atrial appendage (Fig. 3a) and liver (Fig. 3b), and an ill-defined lesion with striped pattern in cerebellar hemisphere were noted.

#### Histology and immunohistochemistry

**Fig. 4:**
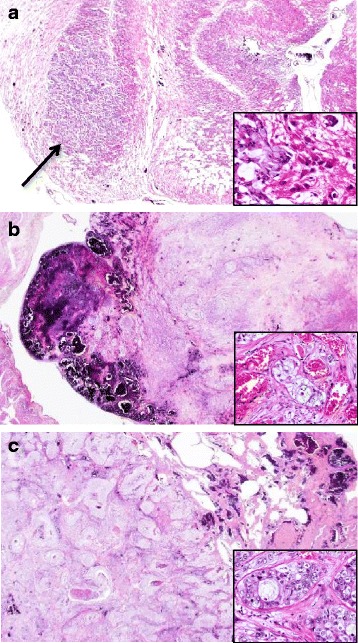
Microscopic findings of tumor-to-tumor metastasis from lung adenocarcinoma. Metastatic adenocarcinoma in cerebellar dysplastic gangliocytoma (**a**) (H.E. x40, *inset* x200). Metastatic adenocarcinoma in a hemangioma of the right atrial appendage (**b**) and liver (**c**) (H.E. x40, *inset* x200)

All tissue samples were fixed in 10 % formalin and embedded in paraffin. Several 4-μm sections were cut from each paraffin block. Hematoxylin-eosin (H.E.) and immunohistochemical stains were performed. Microscopic examination of the H.E. sections revealed lung adenocarcinoma including solid nodules with signet-ring cells (Fig. 2b) and acinar patterns (Fig. 2c). Intra-and/or extracellular mucin was detected by Alcian-Blue staining (not shown). The immunohistochemical examination demonstrated positivity for CK7 and negativity for CK20, while the area of solid pattern was negative for TTF-1 and Napsin A. Alveolar spaces in the bilateral lungs were filed with infiltrated neutrophils. The lung cancer metastasized to the left bronchopulmonary and mediastinal lymph nodes, and infiltrated to the chest wall directly, causing intrathoracic dissemination and generalized metastasis. The histological findings of Cowden’s disease included cerebellar dysplastic gangliocytoma (Lhermitte-Duclos disease), multiple dermal keratosis, oral papillomatosis, gastrointestinal polyposis and lipomatosis, and multiple goiters. In the cerebellar dysplastic gangliocytoma, infiltration of signet-ring cell nests was found (Fig. 4a). In addition, metastatic adenocarcinomas forming solid nests were found in cavernous hemangiomas in the right atrial appendage (Fig. 4b) and liver (Fig. 4c). The metastatic adenocarcinomas showed the almost same morphological and immunohistochemical findings as the lung adenocarcinomas: that is, they were positive for CK7 and negative for CK20, TTF-1 and Napsin A. Although the frequency of such primary lung adenocarcinomas is low [4], no adenocarcinoma was detected in other organs. We therefore concluded that each of the metastatic adenocarcinoma originated from the lung.

## Discussion

We presented an autopsy case of a patient with Cowden’s disease and multiple TTM, and these findings agreed with previous reports that TTM occurred more frequently in patients with hereditary cancer syndrome [3]. To our knowledge, this is the first case report of a patient with Cowden’s disease having multiple TTM from a lung cancer to cavernous hemangiomas and cerebellar dysplastic gangliocytoma. Campbell et al. [1] established the following criteria for the diagnosis of TTM: 1) The presence of two or more distinct tumors must exist; 2) The presence of extravascular metastasis; 3) Exclusion of tumor embolism and of “collision tumors”; and 4) Exclusion of tumors that have metastasized to lymphatic systems that were already involved by generalized lymphatic or hematological malignancy. Pamphlett established three criteria for the diagnosis of TTM [5]: 1) The metastatic nidus must be at least partially enclosed by a rim of histologically distinct primary tumor tissue; 2) The existence of a primary carcinoma must be proven; 3) The metastatic tumor must be demonstrably compatible with primary carcinoma by morphological or immunohistochemical methods. Our case almost fully met both sets of criteria for TTM. The most frequent metastatic donor in TTM is lung cancer, followed by breast cancer [2, 6–8], while renal cell carcinoma and meningioma have been reported as the most common recipient tumors [2, 9]. Recipient tumors have been considered to have certain characteristics that make them favorable sites of metastasis, such as hypervascularity, high glycogen and lipid contents, the high expression of cell adhesion molecules, and a slow growth rate [10–12]. However, it has also been reported that TTM cannot be induced by a vascular-rich environment alone [13]. Thus, it remains to be clarified why certain tumors are more likely than others to be recipients of TTM. In turn, Cowden’s disease is a *PTEN* hamartoma tumor syndrome caused by *PTEN* gene mutation. It is an autosomal-dominant disease characterized by the development of multiple hamartomas in the skin, mucosa, digestive tract, breast, and central nervous system, and presents a high risk for malignant tumors in some organs. The pathological findings of the present case fully satisfied the criteria for Cowden’s disease [14]. Breast cancer, thyroid cancer, and endometrial cancer are the most common malignant neoplasms in Cowden’s disease, and lung adenocarcinoma is rare [15]. Although hemangioma was not included in the diagnostic criteria for Cowden’s disease [14], the revised diagnostic criteria proposed by Pilarski et al. [16] included vascular anomalies in the minor criteria. There have also been some reports of complicating hemangioma in patients with *PTEN* mutation [17–19]. Moreover, loss of PTEN function has been reported to result in enhanced angiogenesis, and it was suggested that patients with Cowden’s disease may experience accelerated growth of any incipient tumors due to enhanced angiogenesis [20]. Thus, we speculated that the hemangiomas in our case may have been caused by Cowden’s disease. TTM is considered to occur more frequently in hereditary cancer syndrome because asymptomatic benign tumors are not treated in hereditary cancer syndrome, and thus are at risk of becoming recipient tumors in the long term. For example, hemangioblastoma is rarely excised in von Hippel-Lindau (VHL) disease because of its slow growth and absence of symptoms: therefore, hemangioblastomas have been considered to be a preferred site for metastasis in VHL disease [3]. However, there has been no report describing TTM in Cowden’s disease, even though it is also a hereditary cancer syndrome. This phenomenon remains to be fully explained. In the present case, however, we speculated that there were three reasons for the multiple TTM. First, the patient had asymptomatic and long-standing neoplastic lesions such as multiple hemangiomas and cerebellar dysplastic gangliocytoma, which may have become preferred sites for metastasizing tumors. Second, lung cancer is the most common donor neoplasm in TTM. Third, it has been reported that TTM could occur as a results of metastasis from lung cancer with more aggressive behavior [13], which is similar to the present case.

## Conclusions

To our knowledge, this is the first reported case of Cowden’s disease with multiple TTM from a lung cancer to cavernous hemangiomas and cerebellar dysplastic gangliocytoma. TTM has been considered a rare phenomenon even though it is a hereditary cancer syndrome. The results of the present autopsy case suggest that more case of TTM in hereditary cancer syndrome might be revealed through careful macroscopic examination and sampling of tumors at autopsy, followed by attentive microscopic examination.

## Consent

Written informed consent was obtained from the patient’s family for the publication of this case report and accompanying images. A copy of the written consent is available for review by the Editor-in-Chief of this journal.

## Abbreviation

TTM: Tumor-to-tumor metastasis
